# Trait-Environment Relationships Reveal the Success of Alien Plants Invasiveness in an Urbanized Landscape

**DOI:** 10.3390/plants10081519

**Published:** 2021-07-26

**Authors:** Reham F. El-Barougy, Mohammed A. Dakhil, Mohamed Abdelaal, Ali El-Keblawy, Louis-Félix Bersier

**Affiliations:** 1 Botany and Microbiology Department, Faculty of Science, Damietta University, Damietta 34518, Egypt; 2 Department of Biology-Ecology and Evolution, University of Fribourg, Chemin du Musée 10, 1700 Fribourg, Switzerland; louis-felix.bersier@unifr.ch; 3 Botany and Microbiology Department, Faculty of Science, Helwan University, Cairo 11790, Egypt; mohamed_dakhil@science.helwan.edu.eg; 4 Department of Botany, Faculty of Science, Mansoura University, Mansoura 35516, Egypt; mohamed_eco@mans.edu.eg; 5 Department of Applied Biology, Faculty of Science, University of Sharjah, Sharjah P.O. Box 27272, United Arab Emirates; akeblawy@sharjah.ac.ae

**Keywords:** homogenization, urbanization, human-made pressures, soil resources, plasticity, alien plants, invasion

## Abstract

Urban areas are being affected by rapidly increasing human-made pressures that can strongly homogenize biodiversity, reduce habitat heterogeneity, and facilitate the invasion of alien species. One of the key concerns in invaded urban areas is comparing the trait–environment relationships between alien and native species, to determine the underlying causes of invasiveness. In the current study, we used a trait–environment dataset of 130 native plants and 33 alien plants, recorded in 100 plots covering 50 urban areas and 50 non-urban ones in an urbanization gradient in the arid mountainous Saint-Katherine protected area in Egypt. We measured eleven morphological plant traits for each plant species and ten environmental variables in each plot, including soil resources and human-made pressures, to construct trait–environment associations using a fourth-corner analysis. In addition, we measured the mean functional and phylogenetic distances between the two species groups along an urbanization gradient. Our results revealed strongly significant relationships of alien species traits with human-made pressures and soil resources in urban areas. However, in non-urban areas, alien species traits showed weak and non-significant associations with the environment. Simultaneously, native plants showed consistency in their trait–environment relationships in urban and non-urban areas. In line with these results, the functional and phylogenetic distances declined between the aliens and natives in urban areas, indicating biotic homogenization with increasing urbanization, and increased in non-urban areas, indicating greater divergence between the two species groups. Thereby, this study provided evidence that urbanization can reveal the plasticity of alien species and can also be the leading cause of homogenization in an arid urban area. Future urban studies should investigate the potential causes of taxonomic, genetic, and functional homogenization in species composition in formerly more diverse urbanized areas.

## 1. Introduction

Urbanization is an important factor that facilitates invasion and can be considered a causative driver for the introduction of alien species [1,2]. In urban areas, anthropogenic modifications of the landcover, such as plant-collection, road construction, and human-made activities, have been implicated as a cause of increasing alien species richness and abundance [3]. This increase could be attributed to the fact that alien species tend to exhibit wide environmental tolerance and high phenotypic plasticity, which may enhance their capacity to survive under changes in environmental conditions [4,5,6,7,8]. For example, variations in temperature ranges and rainfall amount have been considered important factors for the invasion of alien species [9]. Furthermore, alien species abundance is supposed to be promoted by increasing land-use intensity [10,11,12], and thereby, the presence of alien species can be favored when urban cover or human-made pressures increase [13]. However, this is not the case for native species, as intensive urban land use, associated with habitat fragmentation [14,15] and changes in ecosystem functioning [16], decreases the range of the specialized native species pool [17,18,19]. Therefore, a comparative study between native and alien species groups cannot be performed in isolation from the environmental determinants of their distribution.

A possible explanation of why alien species respond differently than natives to changes in the environment is often attributed to the plasticity of alien species traits [7]. A meta-analysis conducted by [6] revealed that invasive species exhibit greater plasticity in functional traits, including size, fitness, and growth rate, than native and non-invasive species. In this sense, plant invasion studies have shown that the alien plant traits that promote alien invasiveness are environment-dependent [6,20,21,22,23]. For example, traits relevant for the success of alien species are usually related to stress tolerance in harsh environments (i.e., specific leaf area, SLA) or tolerance to environmental disturbance in human-made environments (height, seed size) [24,25]. Furthermore, traits related to competitiveness (e.g., height) can be beneficial for invasive species in competitive environments [23]. Thus, the success of alien species in urban areas may also be related to the context-dependence of the trait–environment relationships [26], which increase their potential to invade such areas and may be attributed to urbanization impacts on trait–environment associations [27]. These impacts can subsequently change native community structure and resource availability, leading to a prevalence of disturbed habitats, which can increase the susceptibility of native communities to invasion [28,29].

Alien species may also share similar trait–environment associations with resident native species, which can underpin their successful establishment under homogenous environmental conditions [30]. By contrast, alien species may also exhibit relative dissimilarities to natives, which can support alien species in becoming established in new environments, with less competition with resident native species [20,23]. It is evident that biotic distance plays an important role in the ecological similarity and dissimilarity between alien and native species. For example, if an alien species is closely related to a resident native species, it will be expected to share similar traits and occupy a similar niche as those of the natives, due to the robust effect of environmental filtering [31,32]. This filtering reduces the alien species’ trait range to that of natives [33,34,35,36,37,38,39]. A pattern of aliens co-existing with functionally similar natives is expected, for example, in resource-limited ecosystems. Due to this similarity, alien species may be able to exclude native species and occupy a portion of the functional, and possibly of the phylogenetic, space formerly occupied by the native species, resulting in higher homogenization in native species composition [40,41]. Hence, comparative studies between native and alien populations should be conducted within each environment separately assuming that native and alien species are coexisting under homogeneous environmental conditions.

This study aimed to compare the trait–environment relationships between alien and native plants in urban and non-urban areas in Saint-Katherine Protectorate, an arid protected area in South Sinai, Egypt. These comparative relationships are crucial for understanding the response of alien species to changes in environmental conditions (e.g., resource gradients, urbanization gradients). The objectives of the current study were to (1) compare the responses of the vegetative and reproductive traits of alien and native plant species to soil resources and human-made pressures in urban and non-urban areas; (2) assess the response of the biotic distances between alien and native plant species along an urbanization gradient. We expected significant variation in the trait–environment relationships of alien plant species along the urbanization gradient as an intraspecific divergence and plasticity response to changes in environmental conditions [4]. We also expected similarity between alien and native plant species with increasing urbanization, because often only similar and competitive alien species that are well adapted to urban or human-dominated areas are highly successful alien invaders (see [29,42,43] and are expected to replace a broader range of resident native species).

## 2. Results

**Figure 1:**
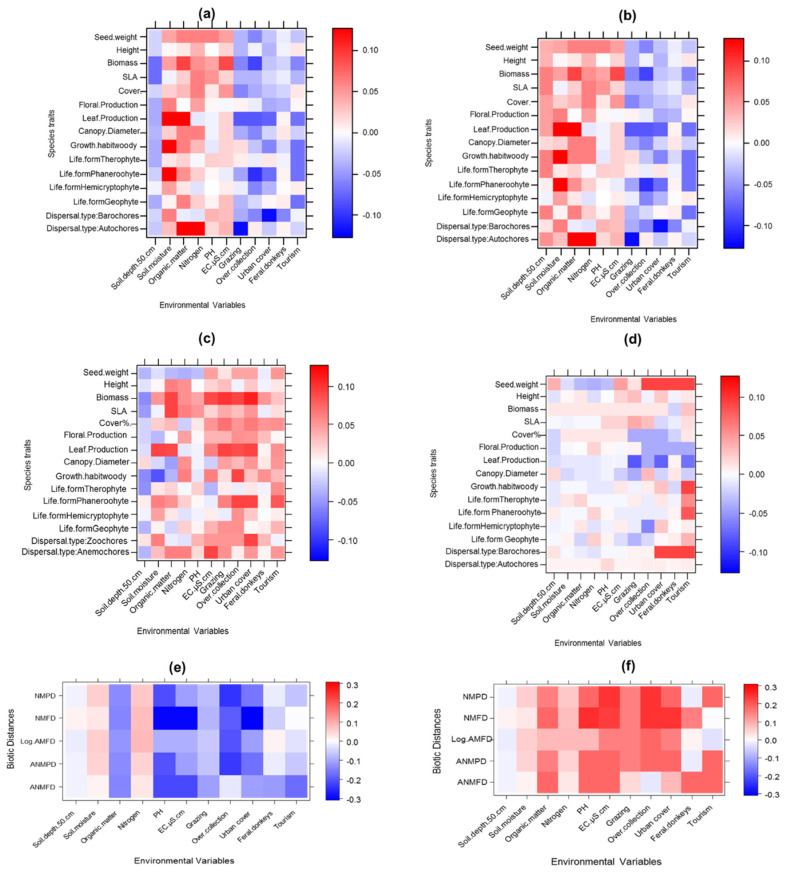
Fourth-corner plots for (**a**) native plants in urban areas (**b**) native plants in non-urban areas (**c**) alien plants in urban areas, and (**d**) alien plants in non-urban areas, (**e**,**f**) the responses of biotic distances between aliens and natives to the environmental variables in urban and non-urban areas, respectively. Figure shows standardized interaction coefficients for plant species traits on (y-axis) and environmental variables on (x-axis). Note: the abbreviations are: NMPD: native mean phylogenetic distance; NMFD: native mean functional distance; AMPD: alien mean phylogenetic distance; AMFD: alien mean functional distance; ANMPD: alien and native mean phylogenetic distance; ANMFD: alien and native mean functional distance; SLA: specific leaf area (cm^2^/gm).

The fourth corner analysis revealed a larger number of strong trait–environment relationships for alien plants in urban habitats compared to non-urban ones (Figure 1a,b). By contrast, native plants showed consistency in their trait–environment relationships in both types of habitats (Figure 1c,d). In parallel, the biotic distances between alien and native plants declined strongly with increasing human-made pressures in urban habitats, while these distances increased in non-urbanized ones (Figure 1e,f). This indicates high biotic homogenization between the two species groups in urban habitats, but more divergence in non-urban ones. 

### 2.1. Trait–Environment Associations in Urban Habitats

First, alien plants were well adapted to urban environmental conditions, which is evidenced by the strong positive relationships between most of their traits and all environmental variables expressing human-made pressures (Figure 1a). Alien plants had higher values of seed mass, SLA, height, leaf, and floral production and shoot biomass with increasing urban cover, grazing, native plant collection, and tourism activity. In addition, alien plants with zoochores and anemochores dispersal types were more frequent and showed more positive relationships with human-made pressures than with soil properties. Second, native plants in both urban and non-urban habitats showed highly significant positive responses to soil properties but negative responses to human-made pressures (Figure 1c). The exception was soil depth; the relationships between native plants’ traits and soil depth were positive in non-urban habitats but negative in urban habitats. Additionally, native plants with barochores and autochores dispersal types were more frequent in these habitats and exhibited more positive responses to soil nitrogen, organic matter, and moisture. However, native plant trait values were affected negatively by increasing intensity of urban cover, grazing, native plant collection, feral donkeys, and tourism activity (Table 1). 

### 2.2. Trait–Environment Associations in Non-Urban Habitats

Alien plant traits showed few associations with the surrounding environmental variables (Table 1 and Figure 1b). There were positive but non-significant relationships between alien plant species traits, soil properties, and human-made pressures. For example, phanerophyte short-distance dispersing (e.g., autochores and barochores) alien plants were frequent and more abundant in areas with greater tourism and urban cover. On the contrary, native plants showed highly significant associations with the surrounding environmental variables, and their trait–environment associations in non-urban areas were relatively similar to in urban ones (Figure 1d).

### 2.3. Biotic Distances along Environmental Gradient 

In highly urbanized habitats (Figure 1e), there were significant negative associations of biotic distances between alien and native species (ANMFD, ANMPD) with the surrounding environmental variables. Interestingly, functional and phylogenetic mean distances within native plants (NMFD, NMPD) and within alien plants (AMFD, AMPD) declined significantly with increasing soil resources and human-made pressures. In other words, alien plants tended to be more similar to natives, conferring more biotic homogenization between the two species groups.

In non-urbanized habitats, there were mostly positive significant associations between the biotic distances and the environmental conditions (Figure 1f). Conversely to high urbanized habitats, mean functional and phylogenetic distances within alien plants, within native plants, and between the two species groups increased significantly with soil resources and human-made pressures (Table 1). This indicates that alien plants tended to be distinct from natives, suggesting more divergence between the two species groups.

## 3. Discussion

The necessity to conduct meta-analysis studies between native and alien species in order to identify the main causes of alien invasiveness success in the context of their trait–environment relationships has been highlighted in the invasion literature [6,44,45,46,47,48]. Our study provides a unique habitat-dependent framework for alien and native plant species along an urbanization gradient. A striking result is the distinct responses of alien versus native plants with regard to trait–environment associations in urban and non-urban habitats. Alien plants traits had weak associations with the environment in non-urban habitats, while these associations were strong in urban habitats. By contrast, native plants revealed consistency in their trait–environment associations along the urbanization gradient. In addition, the biotic distances between the two species groups (aliens and natives) declined with increasing human-made pressures in highly urbanized habitats, indicating biotic homogenization.

### 3.1. Plasticity of Alien Populations in Urban Habitats

The present study revealed significant associations between the traits of alien populations and the environment only in urban habitats. For example, the coefficients of SLA, leaf production, and biomass of alien plants in responding to soil variables were strongly significant (coef = 0.142; *p* < 0.001, coef = 0.324; *p* < 0.01, coef = 0.276, *p* < 0.001 respectively) in urban areas compared to non-urban ones. This may reflect the plasticity of alien plants species to succeed in urban areas through flexible responses to environmental changes, regulated by their growth traits, such as biomass, height, and reproductive traits [49]. Indeed, a comparative study conducted by [7] revealed that alien species have higher phenotypic plasticity than native species when disturbances prevail. This plasticity enables alien species to tolerate stressful conditions and effectively utilize available resources, which results in their widespread distribution and high growth rate [49,50,51,52,53]. One underlying mechanism that could explain this plasticity is the resource fluctuating hypothesis [54,55], which proposes that alien invasiveness success is attributed to increasing resources arising from human-made pressures or from low resource uptake by the resident natives. This hypothesis speculates that resource-demanding alien species are likely to enter high-resource urban habitats correlated with high human-made pressures. These pressures are supposed to increase the content of soil organic matter and nutrients [56] that can facilitate alien invasiveness. By contrast, native species show homogeneity in their associations with the environment in urban and non-urban habitats, indicating their conservative responses towards urbanization. Such responses may nevertheless be beneficial in urban environments with stressful abiotic conditions that negatively affect the survival of native populations (e.g., numerous urban areas are characterized by solid soil surfaces with high dryness and limited resource uptake [57]).

According to recent invasion research, alien populations may accomplish rapid-growth by reallocating below-ground and above-ground biomass, which could be linked to resources fluctuation [58,59,60]. For example, a comparative study by [56] found that the alien plant species *Argemone ochroleuca* optimized biomass allocation and maximized resource utilization in an urbanized habitat [61] in a way that maximized fitness and growth [62,63]. This shift in biomass allocation suggests that intraspecific variation and potential plasticity in plant functional traits can be considered as an adaptive strategy to changes in environmental conditions [64,65]. Furthermore, a long-term study by [66] argued that fast-growing alien plant species are able to boost nutrient acquisition by allocating more resources to their roots. In this sense, we found that alien plants in urban areas had a significantly positive combination between soil depth and productivity of biomass, leaves, and flowers; traits that confer increased biomass allocation and resource acquisition [60,67]. This association between edaphic variables and alien species traits may enable alien plants to exploit enriched soil resources in abandoned fields. However, in non-urban habitats, alien species tended to act as conservative species, with low functional variation and low metabolic costs, correlated with the utilization of a conservative strategy for resource acquisition (e.g., [68,69]).

### 3.2. Responses of Native and Alien Plants to Human-Made Pressures

In the current study, anthropogenic pressures caused alien species to become well-adapted, while natives were not well suited to such pressures. This can be attributed to the ability of alien species to gain a foothold if there are fluctuations in resources. For example, a sudden disturbance can generate a surge of some resources that cannot be immediately exploited by natives, providing aliens the opportunity to succeed [70]. We found that native species were negatively associated with grazing intensity; however, tall alien species producing more leaves and flowers responded positively to grazing. This finding postulates that alien species associated with rapid growth and a grazing tolerant strategy are more prevalent under high rates of grazing intensity [71,72,73]. In this sense, [74] suggested that intensive grazing should favor faster-growing, more palatable plants (i.e., grazing tolerance). It has been found that intense grazing and urbanization contribute to the addition of more litter layers and liberating resources such as high phosphorus and nitrogen that are deposited in the topsoil and enhance nutrient cycling [75]. 

### 3.3. Biotic Homogenization between Alien and Native Plants

The biotic distances within aliens and between alien and native populations declined in highly urbanized habitats. In other words, alien species here tended to be under-dispersed compared to each other and to resident natives. This pattern indicated that the similarity between alien and native plant species is strongly linked to high urbanized habitats. This similarity could be as a result of biotic homogenization in species composition [29,76] or to a decline in the beta diversity of native plant communities [77,78]. This loss of beta diversity is generally associated with the simultaneous local extinction of resident native species and the introduction of alien species and ruderal ones [77,79,80]. In addition, urban landscapes are highly modified and undergo rapid human–urban expansion [76,81,82,83]. This expansion can greatly change the composition dynamics of species [84,85], as well as increase the connection of water bodies spread throughout urban environments [86,87], leading to more homogenization in species composition. Moreover, urban landscapes with fast-growing modifications are expected to be under climatic changes, such as drought stress, longer and drier periods with water shortage, and extremely hot weather conditions during summer time, with more frequent temperature extremes up to 100 °C [88,89]. These harsh conditions lead to the pre-selection for alien species that are well adapted to the harsh urban conditions due to the lack of antagonists or diseases, as well as their wider physiological amplitude and tolerance to the respective local bio-climatic conditions [90]. This preselection could be a major factor in the homogenizing of urban alien species to cope with harsh urban conditions.

## 4. Materials and Methods

### 4.1. Study Area 

The study was carried out in the Saint Katherine Protectorate (SKP) in Egypt’s South Sinai. Gebel Saint Katherine, Gebel Um Shomer, and Gebel Musa are the tallest peaks in the protectorate, which are made up of igneous and metamorphic rocks (about 2642, 2586, 2285 m a.s.l. respectively). The climate of the southern Sinai is arid, with hot, dry summers and chilly winters, and the region receives little rainfall (Appendix A).

### 4.2. Habitat Types, Soil Analysis, and Human-Made Pressures 

In SKP, alien and native populations were surveyed during the spring and summer seasons of 2019 (March to July) in two distinct habitats: (1) wadis (non-urban habitats), (2) roadsides and gardens (urban habitats). First, during the peak of the growing season (spring), we randomly sampled fifty plots (10 m × 10 m) for further soil and vegetation investigations for each habitat. Soil samples were collected randomly at two depths (0–50 cm and >50 cm, three samples per depth) within each plot. Then soil samples were pooled for each depth of every plot. Note that the soil was not always deeper than 50 cm and we created a two-level factor to represent soil depth (0 for soil <50 cm, and 1 otherwise). In the early morning, soil moisture measurements were obtained with a field hygrometer, which measures the volumetric water content in soil. Soil samples were air-dried at room temperature before being dried in an oven at 70 °C and sieved through a 2-mm sieve. Wet combustion with dichromate at 450 °C was used to determine soil organic matter (OM) [91]. For the estimation of soil electrical conductivity (EC) and pH, soil water extracts (1:5) were made [91]. The Kjeldahl method was used to determine soil nitrogen, as described by Bremner and Mulvaney [92]. 

Second, we estimated the human-made pressures in the studied plots for each habitat, including (1) grazing, (2) tourism, (3) native plant collection, (4) feral donkeys, and (5) urban-cover, since these pressures influence the growth of aliens and natives in natural ecosystems [93]. We ranged these pressures based on their intensity into three levels (zero, low, and high). Together, these five factors created a gradient expected to indicate the degree of disturbance that can potentially favor the establishment of alien species [94], (Appendix A; Table S1).

### 4.3. Traits Measurements

We measured six non-destructive morphological traits for alien and native individuals in each habitat and for each sampled plot at the end of the flowering–fruiting period including: plant height, leaf production, floral production, canopy diameter, cover percentage, and seed weight. In addition, we measured two destructive traits for alien individuals, shoot biomass (kg) and specific leaf area (SLA), directly from the field surveys. For SLA, we scanned the leaves and measured the total leaf area using IMAGEJ software, version 1.49. Then we estimated the SLA by dividing the leaf area by the leaf weight (dry leaf weight) [95]. All aboveground parts (leaves and stems) of all alien plants were dried in a drying oven (VWR International) at 50 °C for three days to obtain measurements for the shoots and then weighed using a Mettler Toledo ML Series Precision Balance (ML Analytical balance). For native plants, we estimated those two destructive traits using the same destructive and non-destructive traits on individuals of the same native species that grew outside their protected range, as explained in [96]. These plant traits were selected according to [97], in order to assess the functional response of both alien and native species to the environmental factors within different habitats. In addition, life forms, growth habit (woody, non-woody), and dispersal types were recorded for each studied species (alien and native). 

### 4.4. Biotic Distances 

Within each plot, we calculated the mean pairwise phylogenetic distances within native plants (NMPD), within alien plants (AMPD), and between alien and native plants (ANMPD) [98] (Appendix A; Table S2). To quantify these distances, we used four commonly sequenced genes available in GenBank [99]: rbcL, matK, ITS1, and 5.8 s to build a phylogenetic tree of the 166 species (33 aliens, 130 natives). At least one gene from each of the 130 native species was found in GenBank. We used known sequences from congeneric species as a surrogate for the 10 native species that lacked sequencing data (see phylogenetic guidelines by Jin and Cadotte 2015). As an outgroup species, we included the genetic sequence of Amborella trichopoda Baill. because it diverged early in angiosperm evolution. The methods used to generate the phylogeny are previously described in [100]. Appendix A; Figure S1 provides the final ultrametric phylogenetic tree, containing all 130 native, and 33 alien, species found in this study. Then, the phylogenetic distances were calculated using the function MPD in the R package “picante” version 1.8 [101]. Within each plot, we also measured the mean functional distance among natives (NMFD), among aliens (AMFD), and between alien and native plant species (ANMFD) using a set of measured traits (height, SLA, biomass, leaf production and floral production, seed weight). NMFD, AMFD, and ANMFD were calculated as the mean weighted (by abundance) pairwise Euclidian distance between each pair of species in the corresponding group within each plot [69,96,102], using the ‘dist’ function in R package ‘stats’ (version 3.7.0). 

### 4.5. Fourth Corner Analysis

Since we aimed to study the relationship of species traits to the environmental conditions by considering the abundances of species in the studied plots, we used three matrices: species abundance matrix (S), environmental matrix (E), and trait matrix (T). Then, these matrices were compiled directly for each species group using a fourth corner approach, as implemented in the function *traitglm* of package mvabund in R [103]. The fourth corner analysis compiles *S* (first upper-left-corner, representing the abundance of each plant species (columns) across all plots (rows)), *E* (second upper-right-corner, representing the environmental variables (columns) across all plots (rows)), and *T* (third lower-left-corner, representing the trait values (rows) for all species (columns)), to estimate the fourth unknown lower-right-corner matrix that explains the trait–environmental correlations [104]. We also investigated the collinearity between environmental variables and ruled out variables with r > |0.7| [105]. Finally, we calculated the coefficient estimates of all explanatory environmental variables using the *manyglm* function [106]. Coefficients describe how environmental variables can predict changes in traits. In addition, we used the function *anova.traitglm,* based on bootstrapping with 999 permutations, to test for the statistical significance of trait–environment relationships in predicting the presence of alien and native species in the study area.

## 5. Conclusions

Our results provide evidence that urbanization can reveal the plasticity of alien species in an arid protected area and that can drive alien invasiveness. Urbanization can also be the cause of the biotic homogenization between alien and native populations in SKP. This similarity may be due to the low beta-diversity and weak spatial variation in the structure and composition of resident communities. However, the smaller biotic distances between alien and native species could be early signs of homogenization associated with urbanization. Although we were able to reveal some interesting comparative patterns between alien and native species that were associated with urbanization intensity, further investigation is needed to identify all the potential factors underlying these patterns. For instance, it would be necessary to evaluate how invasion history, disturbance history, site age, land-use legacies, and microclimatic conditions are related to beta diversity patterns, as these factors have previously been shown to be essential for explaining homogeneity in species composition in urban lands (e.g., [107,108,109]). Correspondingly, urbanization as a driver of biotic homogenization of formerly distinct regional biota would be a topic of increasing concern for future urban studies.

## Figures and Tables

**Table 1:** Results of fourth corner analysis using 999 permutations (probability integral transform residual bootstrap (PIT-trap) block resampling, which accounts for correlation) for alien and native plant groupings, as well as biotic distances between the two species groups.

Urbanized Areas					Non-Urbanized Areas				
env:trait	Res.Df	Df.diff	Dev	Pr	env:trait	Res.Df	Df.diff	Dev	Pr
(Fourth corner)					(Fourth corner)				
**Alien species**	3086	66	163.8	0.01 **	**Alien species**	1255	43	58.2	0.09
**Native species**	5022	87	268.3	0.01 **	**Native species**	5344	98	276.6	0.01 **
**Biotic distances**	665	60	86.08	0.01**	**Biotic distances**	236	60	101.4	0.01 **

Note that abbreviations are DF: degrees of freedom; Df.diff: difference in degrees of freedom; Res.df: residual degrees of freedom or change in degrees of freedom; Dev: deviation; Pr: p values (significance). Note: ** refers to significance.

## Data Availability

Not applicable.

## Supplementary Materials

The following are available online at https://www.mdpi.com/article/10.3390/plants10081519/s1, Figure S1: Rooted ultrametric phylogenetic tree including the pool of 130 native species and 33 alien species collected from 83 pairs of invaded and non-invaded plots in Saint Katherine Protectorate, Table S1: shows the description of all the studied variables, Table S2: shows the description of all abbreviations that were stated in the studied work. Reference [110] is cited in the Supplementary Materials.

## Appendix A

Study area characteristics: The study area was conducted in Saint Katherine Protectorate (SKP), South Sinai, Egypt (Figure 1a), from March to July 2018 at the peak of the flowering season (Danin 2006). Saint Katherine Protectorate (SKP) was declared in 1996 as full protected-area status was given to approximately 4350 km^2^ of largely mountainous terrain in South Sinai, but the studied area was given to approximately 100–150 km^2^. The area includes the highest peaks in Egypt and contains a unique assemblage of natural resources, notably high-altitude ecosystems with surprisingly diverse fauna and flora and with a significant proportion of endemic species. The high mountains (1600–2460 m a.s.l.) surrounding the town of St Katherine receive higher levels of precipitation, of up to 100 mm per year [111].

Climate: The Saint Katherine Protectorate lies in the arid North African belt and is characterized by a Saharan-Mediterranean climate, experiencing extremely dry, hot summers and cold winters. Average rainfall is 57 mm a year, with maximum temperatures of 35 °C and lows of 5 °C. The high mountains (1600–2460 m a.s.l.) surrounding the town of St Katherine receive higher levels of precipitation, of up to 100 mm per year [111]. SKP encompasses approximately 4350 km^2^ of largely mountainous terrain in the South Sinai Governorate, but the study area was approximately 100–150 km^2^ in size.

Native destructive trait data: As destruction of native biodiversity is strictly forbidden within the SKP, we could not harvest the leaves or any other part of the native species. Therefore, we measured the total leaf area of the plants, by first drawing the outlines of their leaves on paper, and then measuring the areas of these leaf copies. We then measured the same traits, as well as SLA and aboveground biomass, on individuals of the native species that grew outside the protected areas. Then for each native species, we used multiple regression models to determine allometric equations [94] for SLA and aboveground biomass as functions of the non-destructive measurements. We then used these allometric equations, and the non-destructive measurements that we took in the SKP plots to estimate the SLA and aboveground biomass of the native plants there
