# Supplementary material for: Trait-Environment Relationships Reveal the Success of Alien Plants Invasiveness in an Urbanized Landscape
Source: Plants (Basel). 2021 Jul 26;10(8):1519. doi: 10.3390/plants10081519 (PMC8399185; doi:10.3390/plants10081519)
Supplement: Supplementary file 1 [file plants-10-01519-s001.zip › plants-1292058-supplementary.pdf]

Supplementary Material

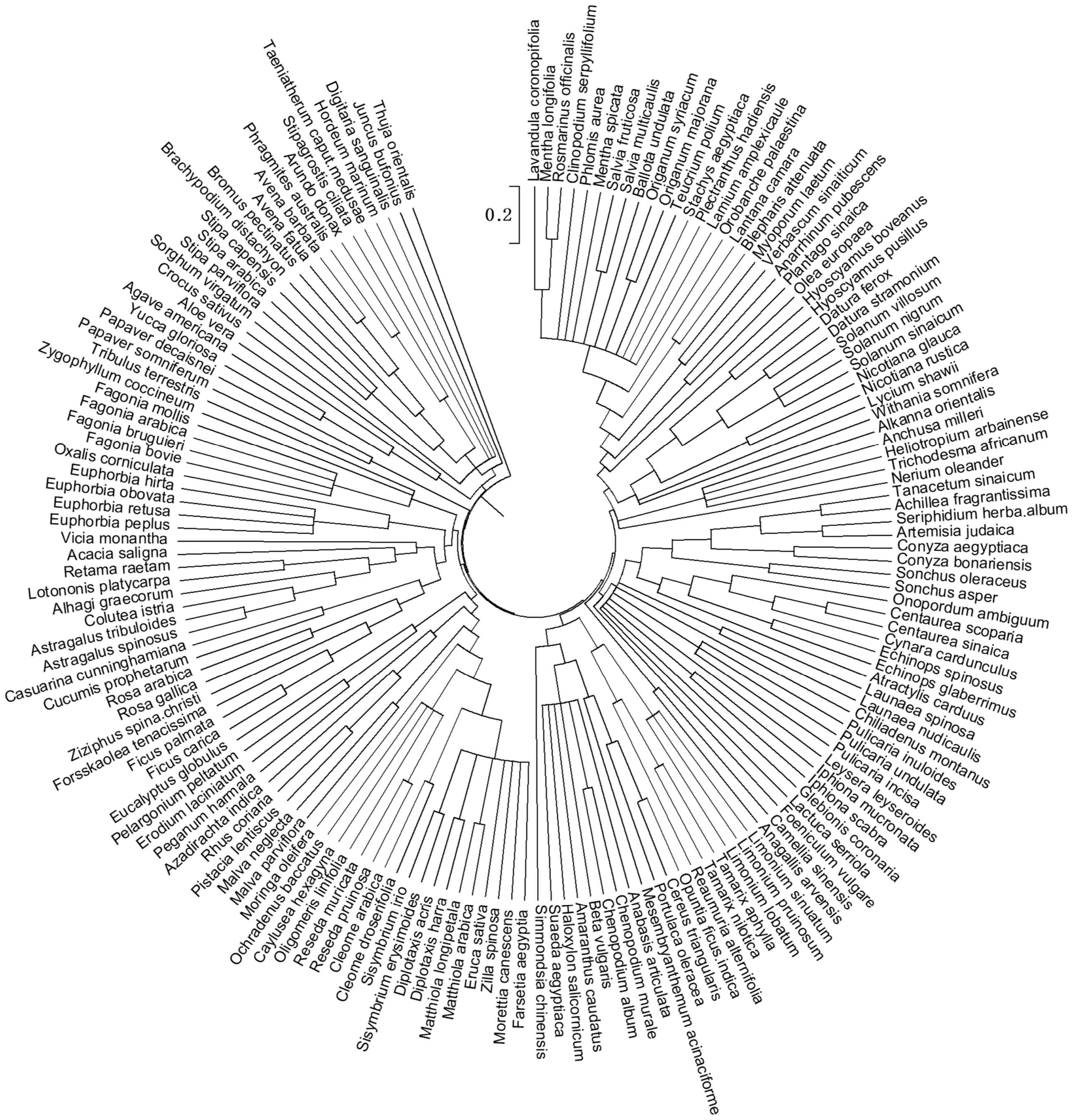

Figure S1. Rooted ultrametric phylogenetic tree including the pool of 130 native species and 33 alien species collected from 83 pairs of invaded and non-invaded plots in Saint Katherine Protectorate. This tree was created by El-Barougy, Reham.F using PhyML-3.0 software ,version 3.0 [110].

Table S1. shows the description of all the studied variables.

Variable Category	Variables	biotic / abiotic	Value	Unit/Description	Source
Plant Species Traits	Height	biotic	metric	cm	Field survey
	SLA	biotic	metric	cm2/gm	Lab analysis
	Number of leaves	biotic	metric	integer number	Field survey
	Canopy Diameter	biotic	metric	cm	Field Survey
	Number of flowers and fruits	biotic	metric	integer number	Field survey
	Above-ground-bio-mass	biotic	metric	gm	Lab analysis
Edaphic varia-bles	Organic matter	abiotic	metric	percentage	Lab analysis
	Nitrogen	abiotic	metric	mg/L	Lab analysis
	Soil Depth	abiotic	Factor (2 levels of soil depth:0-50 and > 50) 1-	Zero -low depth, high depth	
	Soil moisture	abiotic	metric	percentage	Lab analysis
Anthropo-genic pres-sures	Grazing	biotic	1-3 values for three different levels of pressure intensity	1- high level, 2- me-dium level, 3-high level	Field survey
	Tourism	biotic			Field survey
	Urban cover	biotic			Field survey
	Over-collection	biotic			Field survey

Table S2. shows the description of all abbreviations that were stated in the studied work.

Acrynom	Description
**SKP**	*Saint Katherine Protectorate*
**NMPD**	*Native Mean Phylogenetic Distance*
**NMFD**	*Native Mean Functional Distance*
**AMPD**	*Alien Mean Phylogenetic Distance*
**AMFD**	*Alien Mean Functional Distance*
**ANMPD**	*Alien and Native Mean Phylogenetic Distance*
**ANMFD**	*Alien and Native Mean Functional Distance*
**SLA**	*Specific Leaf area (cm2/gm)*
